# Identification of diagnostic immune-related gene biomarkers for predicting heart failure after acute myocardial infarction

**DOI:** 10.1515/med-2023-0878

**Published:** 2023-12-21

**Authors:** Yingchun Hu, Xiaoyu Chen, Xiyuan Mei, Zhen Luo, Hongguang Wu, Hao Zhang, Qingchun Zeng, Hao Ren, Dingli Xu

**Affiliations:** State Key Laboratory of Organ Failure Research, Department of Cardiology, Nanfang Hospital, Southern Medical University, Guangzhou, 510515, Guangdong, China; Department of Nephrology, Rheumatism and Immunology, Chongqing Jiulongpo People’s Hospital, Chongqing, 400050, China; Department of Arrhythmic, Cardiovascular Medical Center, Shenzhen Hospital of University of Hong Kong, Shenzhen, 518040, Guangdong, China; Department of Cardiology, Nanfang Hospital, Southern Medical University, Guangzhou, 510515, Guangdong, China; Department of Rheumatology, Nanfang Hospital, Southern Medical University, Guangzhou, 510515, Guangdong, China

**Keywords:** IL17 signaling pathway, NF-κB signaling pathway, immune infiltration, immune-related biomarkers

## Abstract

Post-myocardial infarction heart failure (HF) is a major public health concern. Previous studies have reported the critical role of immune response in HF pathogenesis. However, limited studies have reported predictive immune-associated biomarkers for HF. So we attempted to identify potential immune-related indicators for HF early diagnosis and therapy guidance. This study identified two potential immune-related hub genes (IRHGs), namely *CXCR5* and *FOS*, using bioinformatic approaches. The expression levels of *CXCR5* and *FOS* and their ability to predict long-term HF were analyzed. Functional enrichment analysis revealed that the hub genes were enriched in immune system processes, including the interleukin-17 and nuclear factor-kappa B signaling pathways, which are involved in the pathogenesis of HF. Quantitative real-time polymerase chain reaction revealed that the *Fos* mRNA levels, but not the *Cxcr5* mRNA levels, were downregulated in the mice of the HF group. This study successfully identified two IRHGs that were significantly and differentially expressed in the HF group and could predict long-term HF, providing novel insights for future studies on HF and developing novel therapeutic targets for HF.

## 1 Introduction

Inflammation is involved in the pathogenesis of heart failure (HF). Under stress conditions, cardiomyocytes release proinflammatory cytokines [1], which trigger immune responses (including macrophage infiltration into the heart [2]) and mediate disease progression [3]. T cells are reported to mediate the progression of pressure overload-induced HF [4,5,6]. Additionally, the immune response in the impaired myocardium is characterized by the activation of the classical human immune response, which is similar to the response to autoimmunity and infection [7]. These findings indicate the potential correlation between myocardial diseases, cytokines, and immune cell populations. Previous studies have demonstrated the role of immune response in HF pathogenesis [8]. However, limited studies have identified the immune-associated biomarkers for predicting HF. So we attempted to identify potential immune-related indicators for HF early diagnosis and therapy guidance.

Recently, bioinformatics approaches, a type of machine learning, have been used to identify biomarkers for the diagnosis and therapy guidance of HF [9,10]. Cell-type identification by estimating relative subsets of RNA transcripts (CIBERSORT), a widely used algorithm for identifying immune cells, enables the assessment of relevant subpopulations of RNA transcripts and the quantification of cell components [11]. Additionally, CIBERSORT enables the elucidation of the immune cell landscape in tumors [12,13].

Bioinformatics approaches have identified several candidate genes for post-acute myocardial infarction (MI) (AMI) HF. However, the immune mechanisms of these genes in post-AMI HF have not been elucidated. Maciejak et al. [14] revealed potential biomarkers for predicting post-AMI HF, including *FMN1*, *JDP2*, and *RNASE1*. Similarly, Li et al. [15] discovered candidate time series differentially expressed genes (DEGs) associated with post-AMI HF, namely *FADS2*, *LRRN3*, *GPR15*, and *AK5*.

This study aimed to identify immune-associated biomarkers to complement the known predictive indicators [14,15] and reveal the differential immune cell infiltration statuses between the HF and non-HF groups. Additionally, this study explored the diagnostic value of the identified biomarkers to provide novel insights for future studies on HF and develop novel therapeutic targets for HF.

## 2 Materials and methods

### 2.1 Data acquisition

The microarray expression dataset GSE59867 was downloaded from the Gene Expression Omnibus (www.ncbi.nlm.nih.gov/geo) database using the “BiocManager” package in R. The GSE59867 dataset was developed based on the GPL6244 platform ([HuGene-1_0-st] Affymetrix Human Gene 1.0 ST Array [transcript (gene) version]). The dataset comprised the data of 111 patients with ST-elevation MI at four different time points (admission, the day of AMI diagnosis; discharge, days 4–6 post-AMI; month 1 post-AMI; and month 6 post-AMI). The control group comprised 46 patients who were diagnosed with stable coronary artery disease on the day of admission. After 6 months, 9 patients were considered to exhibit HF and 8 patients did not exhibit HF according to the first and fourth quartiles of plasma N-terminal pro-B type natriuretic peptide (NT-proBNP) level and left ventricular ejection fraction. The demographic characteristics, including age, sex, body mass index, medical history, drug administration, and smoking history, were not significantly different between the HF and non-HF groups (*p* > 0.05). Immune-related genes (IRGs) were downloaded from the ImmPort database (https://www.immport.org/home).

### 2.2 Data pre-processing

The GSE59867 raw dataset was subjected to pre-processing methods, including background calibration, normalization, and log_2_ transformation, using the “Affy” package [16] in R “Bioconductor.” Based on the probe annotation information, the probes were converted into gene symbols. If multiple probes corresponded to the same gene, the median value was considered its gene expression value. Additionally, the probes matching with more than one gene were removed.

### 2.3 DEG analysis

DEGs between the HF and non-HF groups were screened using the “Limma” package [17] in R based on the following criteria: *p* < 0.05 and |log_2_-fold change (FC)| > 0.5. DEGs with log_2_FC > 0.5 were considered upregulated genes, whereas those with log_2_FC < −0.5 were considered downregulated genes.

### 2.4 Gene ontology (GO) and Kyoto Encyclopedia of Genes and Genomes (KEGG) pathway enrichment analyses and gene set enrichment analysis (GSEA)

The potential functions of DEGs were evaluated using GO and KEGG pathway enrichment analyses with the “ClusterProfiler” package [18] in R language. The enrichment of GO terms and KEGG pathways was considered significant at *p* < 0.05. Functional enrichment of DEGs was further examined using GSEA as previously reported [19]. GSEA was performed using the “ClusterProfiler” package in R language and the enrichment was considered significant based on the following criteria: |normalized enrichment score| > 1; *p* < 0.05; false discovery rate < 0.25. Gene symbols were converted to Entrez ID using the human genome annotation package “org.Hs.eg.db.” and plotted using the “ggplot2” package [20] in R.

### 2.5 Protein–protein interaction (PPI) network construction and module analysis

STRING software (version 11.5) was used to construct the network of DEGs (|log_2_FC| > 0.5 and *p* < 0.05) with the combined score of >0.4 considered the cut-off criterion. The PPI network was imported into Cytoscape software (version 3.8.2) for further visualization. The Molecular Complex Detection (MCODE) plugin (version 2.0.0) in Cytoscape software was used to identify the most significant module. The operating parameters were as follows: degree cut-off = 2; node score cut-off = 0.2; *k*-score = 2; and max. depth = 100. The top 10 hub genes were identified using the CytoHubba plugin (version 0.1) in Cytoscape.

### 2.6 Estimation of infiltrating immune cell abundance

The CIBERSORT algorithm enables the identification of various immune cells based on their expression profiles [11]. The normalized GSE59867 expression matrix was analyzed using the CIBERSORT algorithm to obtain the proportions of various immune cell types. The core algorithm of CIBERSORT is the deconvolution algorithm, which utilizes the single-cell gene expression profiles to compute the proportion of different cell types.

### 2.7 Receiver operating characteristic (ROC) curve analysis

The sensitivity and specificity of the hub genes to distinctly identify post-AMI HF were determined using ROC curve analysis. The pROC package in R language was used to obtain the sensitivity, specificity, and area under the ROC curve (AUC) values.

### 2.8 Quantitative real-time polymerase chain reaction (qRT-PCR)

Total mRNA was extracted using TRIzol reagent and reverse-transcribed into complementary DNA using the Evo Maloney murine leukemia virus reverse transcription Premix for qPCR (Accurate Biotechnology, Hunan, China). qRT-PCR analysis was performed using SYBR^®^ Green Pro Taq HS Premix (Accurate Biotechnology, Hunan, China) and LightCysler480 Real-Time PCR System (Germany). The primers for *Fos*, *Cxcr5*, and *Actb* were synthesized by Tsingke Biology (Beijing, China). The gene expression levels were analyzed using the 2^−ΔΔCt^ method. The expression levels of target genes were normalized to those of *Actb*. The primers used in qRT-PCR analysis are listed in Table 1.

**Table 1:** Primer sequences

Gene	Forward (5′–3′)	Reverse (5′–3′)
*Fos*	GAGGAGGGAGCTGACAGATACACT	GATTGGCAATCTCAGTCTGCAA
*Cxcr5*	TGGCCTTCTACAGTAACAGCA	GCATGAATACCGCCTTAAAGGAC
*Actb*	CTACCTCATGAAGATCCTGACC	CACAGCTTCTCTTTGATGTCAC

### 2.9 Western blotting

The samples were lysed using a mixture of radioimmunoprecipitation assay lysis buffer, phosphatase inhibitor, and phenylmethylsulfonyl fluoride (100:1:1) and an Ultrasonic Cell Disruptor. The lysates were subjected to electrophoresis. The resolved proteins were transferred to a polyvinyl difluoride membrane (Millipore, USA). The membrane was blocked with 5% milk powder in Tris-buffered saline containing Tween-20 (TBST) for 1 h. Next, the membrane was probed with the anti-ANP (1:1,000, Proteintech, China) antibodies at 4°C overnight, followed by incubation with the horseradish peroxidase-labeled secondary antibodies at room temperature for 1 h. After washing the membrane thrice with TBST, immunoreactive signals were developed using an enhanced chemiluminescence reagent (Fude Biological, Hangzhou, China).

### 2.10 Animal model establishment

C57BL/6 mice (22–25 g) obtained from the Animal Center of Nanfang Medical University were randomly divided into the sham (*n* = 5) and MI (*n* = 5) groups. Mice in the sham group underwent sham surgery, while those in the MI group were subjected to left anterior descending artery permanent ligation as described previously [21]. Animals were maintained under the following conditions for 2 months: circadian cycle, 12 h light/dark cycle; temperature, 22°C; access to water and food, ad libitum. Mice were then anesthetized and sacrificed.

### 2.11 Masson’s staining

The paraffin heart sections were deparaffinized, rehydrated, and subjected to Masson’s staining with the Masson’s kit (# G1340; Solarbio), following the manufacturer’s instructions. The sections were sealed and observed.

### 2.12 Statistical analysis

All statistical analyses were performed using GraphPad Prism (version 8.0.2) and R software (version 4.2.4). Data are presented as mean ± standard error of the mean. The sensitivity and specificity of hub genes to distinguish post-AMI HF from post-AMI non-HF were calculated and represented using an ROC curve. All tests in this study were two-tailed. Differences were considered significant at *p* < 0.05.


**Ethics approval and consent to participate:** Animal experiments were performed according to the instructions of the Animal Ethics Committee of Nanfang Medical University and the ARRIVE guidelines. Efforts were made to minimize animal suffering.

## 3 Results

### 3.1 Identification of DEGs between the non-HF and HF groups

**Figure 1:**
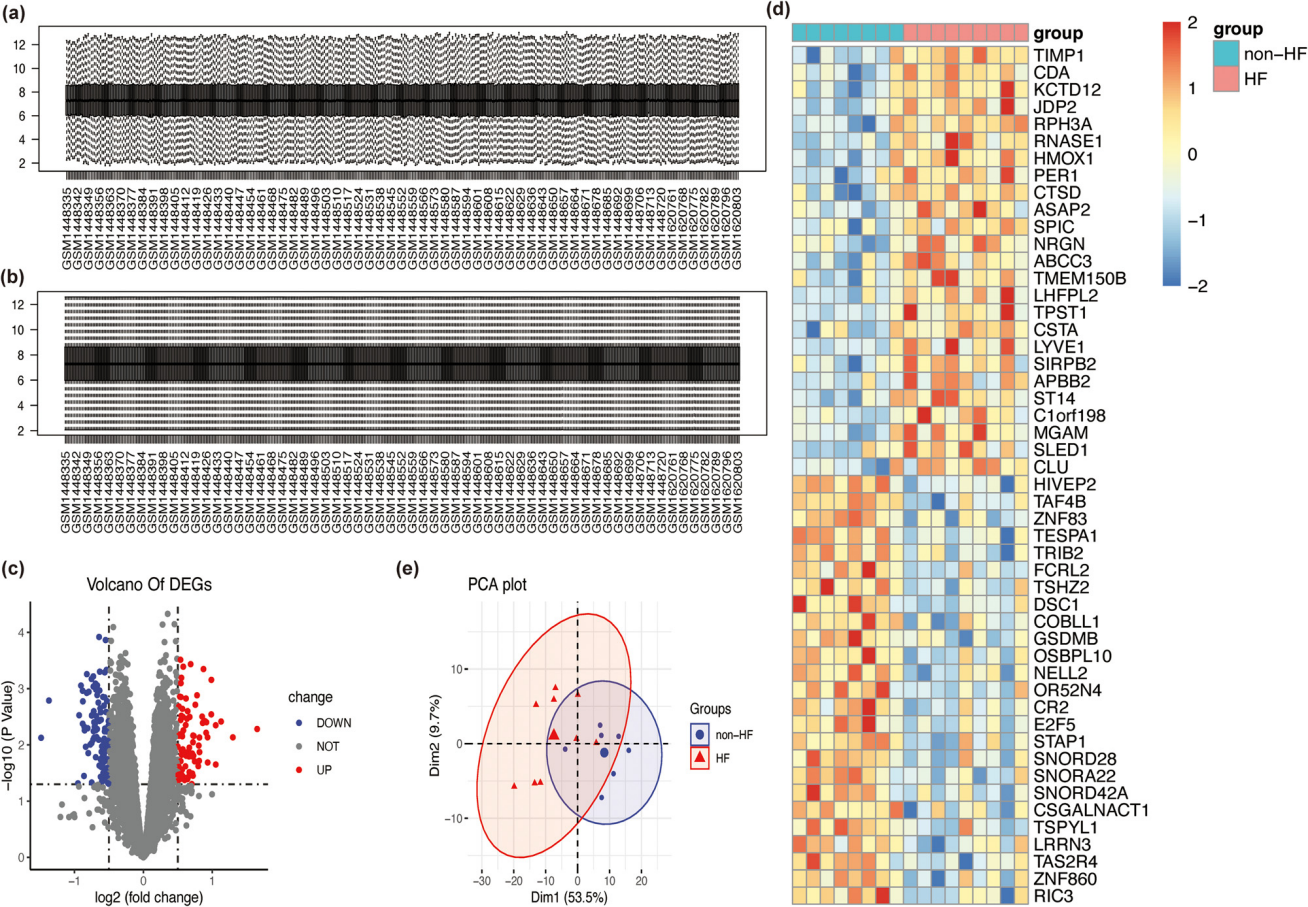
(a) The expression values before normalization. (b) The expression values after normalization. (c) The volcano map of DEGs (red and blue dots represent upregulated and downregulated genes, respectively). (d) The heatmap of the DEGs (each row represents one DEG, while each column represents one sample. The red and blue colors indicate upregulated and downregulated DEGs, respectively). (e) Principal component analysis of DEGs between the HF and non-HF groups revealed that the two groups were separated in the Dim1 component.

To identify the critical genes involved in the pathogenesis of post-AMI HF, the GSE59867 dataset was analyzed, which revealed nine patients with HF and eight non-HF cases whose samples were collected at admission. The gene expression values in the GSE59867 dataset were normalized (Figure 1a and b). In total, 200 DEGs were identified between the non-HF and HF groups (84 upregulated genes and 116 downregulated genes) (Figure 1c). The heatmap of DEGs was generated, which revealed a distinct expression pattern between the non-HF and HF groups (Figure 1d). To further assess the differential gene expression patterns between the HF and non-HF groups, the GSE59867 dataset was subjected to principal component analysis. The two groups were distinctly separated in the Dim1 component (Figure 1e). These findings indicate the well-replicated data between the HF and non-HF groups.

### 3.2 Functional enrichment analysis of DEGs

**Figure 2:**
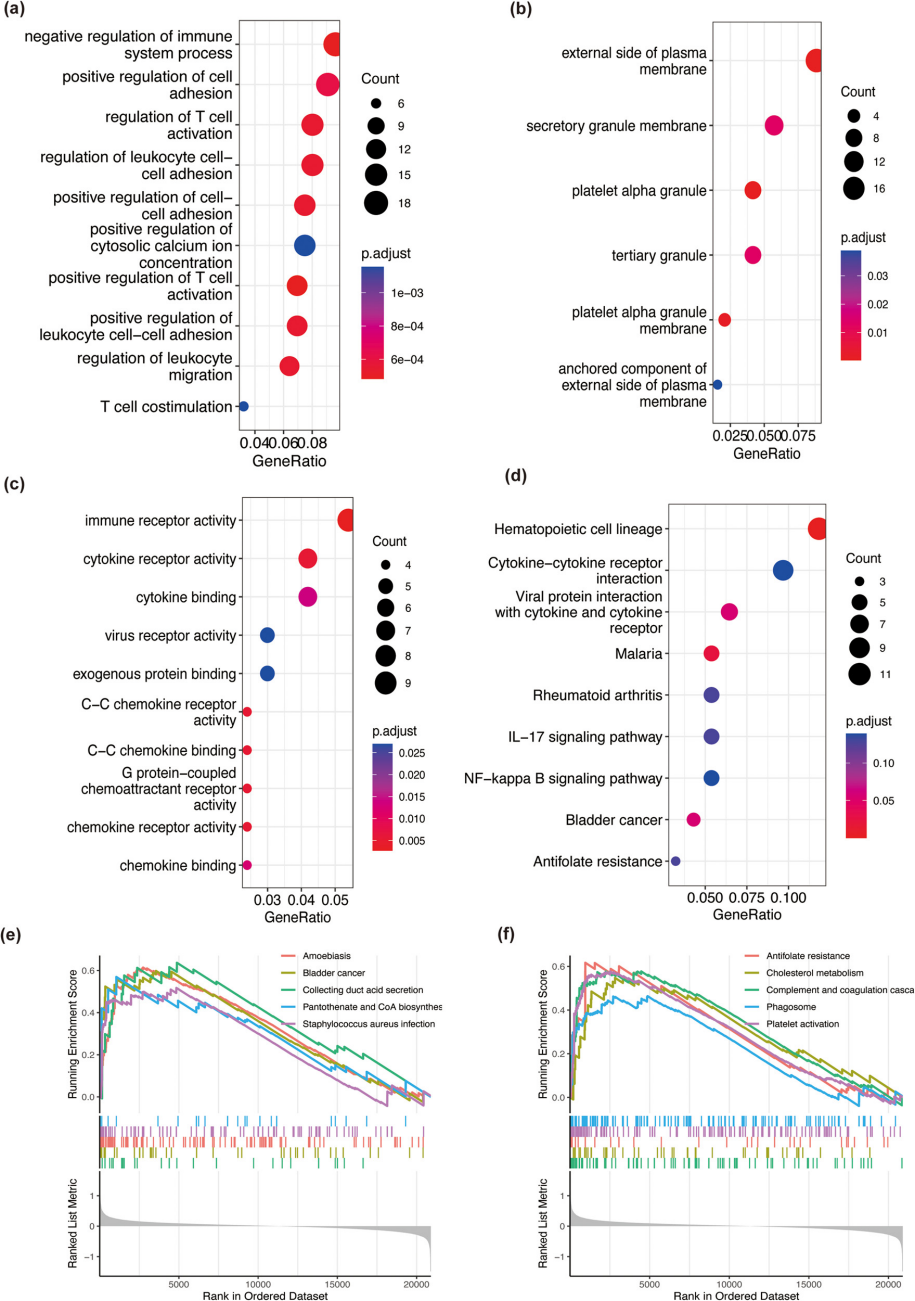
GO and KEGG pathway enrichment analyses and GSEA of DEGs. The red and blue colors represent high and low adjusted *p* values, respectively. The size of the dot indicates the amount of enriched DEGs. (a) The top 10 BP enrichment terms. (b) The top 10 CC enrichment terms. (c) The top 10 MF enrichment terms. (d) Enriched KEGG pathways. (e and f) The top 10 enriched pathways were identified using GSEA.

To examine the functions of the 200 identified DEGs, the DEGs were subjected to GO and KEGG pathway enrichment analyses using the “ClusterProfiler” packages in R language. The DEGs were mainly enriched in various GO terms as follows: biological process (BP) term (Figure 2a), immune system process; cellular component (CC) term (Figure 2b), external side of plasma membrane; molecular function (MF) term (Figure 2c), immune receptor activity, cytokine receptor activity, and cytokine binding. KEGG pathway (Figure 2d) enrichment analysis revealed that the DEGs were enriched in hematopoietic cell lineage.

GSEA revealed that the top 10 enriched pathways in which the DEGs were enriched included amoebiasis and pantothenate and CoA biosynthesis (Figure 2e and f).

### 3.3 Potential key IRGs associated with HF

**Figure 3:**
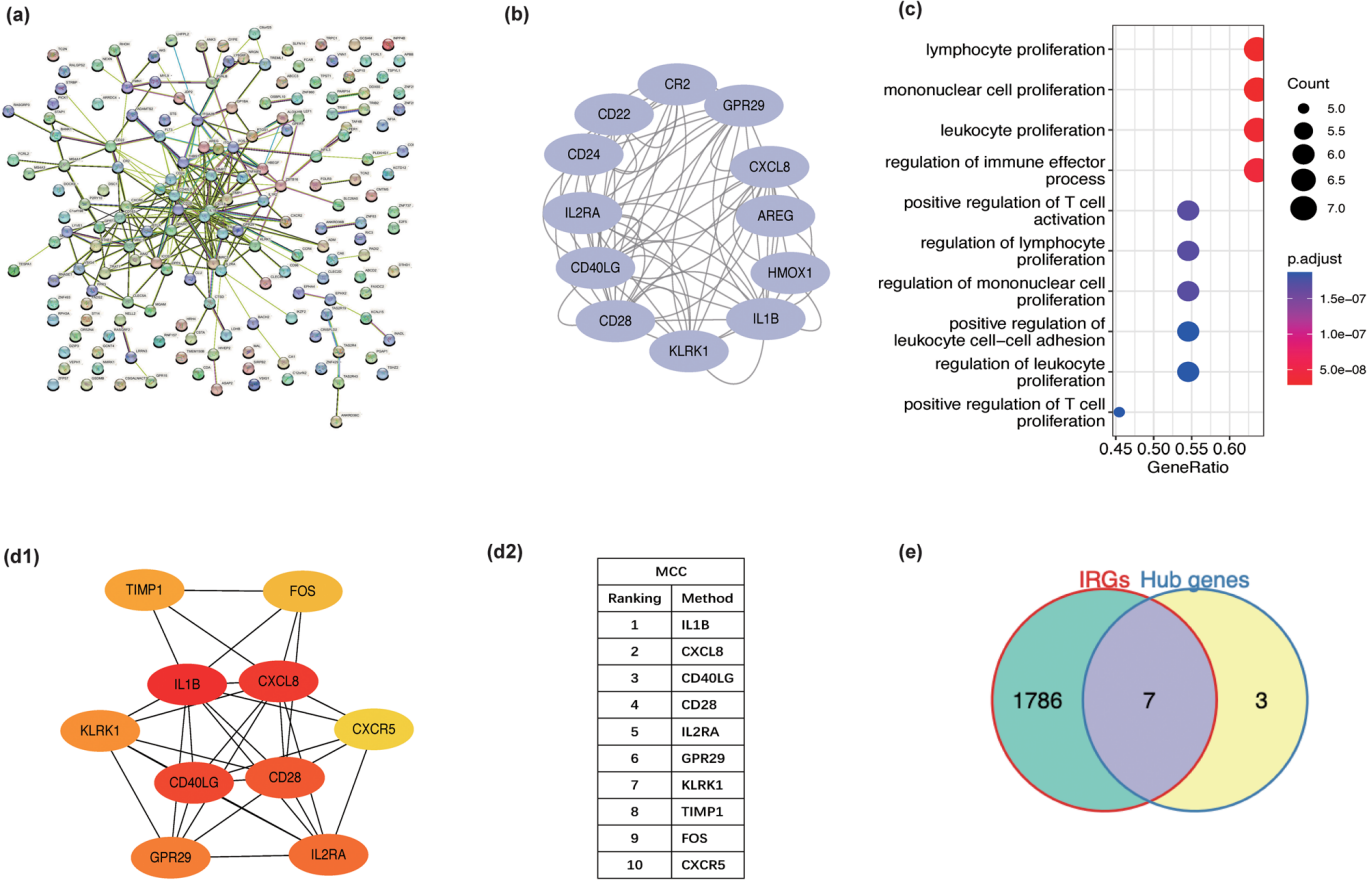
(a) The PPI network of DEGs was constructed using the STRING online tool. (b) The first module obtained using the Cytoscape software and MCODE plugin. (c) Enrichment analysis of the first module. (d1 and d2) The top 10 hub genes among DEGs were identified using the CytoHubba plugin in Cytoscape software. (e) Venn diagram shows overlapping genes between hub genes and IRGs.

To examine the interaction between the identified DEGs, a PPI network was constructed using the STRING online tool (Figure 3a). The PPI network was imported into the Cytoscape software for further analysis. Module analysis was performed using the MCODE plugin to obtain the first module, which was subjected to subsequent analysis (Figure 3b). The first module was enriched in lymphocyte proliferation (Figure 3c). To identify the hub genes among the DEGs, the CytoHubba plugin in Cytoscape software was used to extract the top 10 hub genes (*IL1B*, *CD28*, *CXCL8*, *IL2RA*, *KLRC4-KLRK1*, *CXCR5*, *CD40LG*, *FOS*, *TIMP1*, and *CCR6*) (Figure 3d1 and d2).

Functional enrichment analyses revealed that the DEGs were enriched in immune responses. Hence, the IRGs were downloaded from the ImmPort database. The intersection of 10 hub genes and IRGs revealed seven immune-related hub genes (IRHGs) (Figure 3e).

### 3.4 Functional enrichment analysis of IRHGs

**Figure 4:**
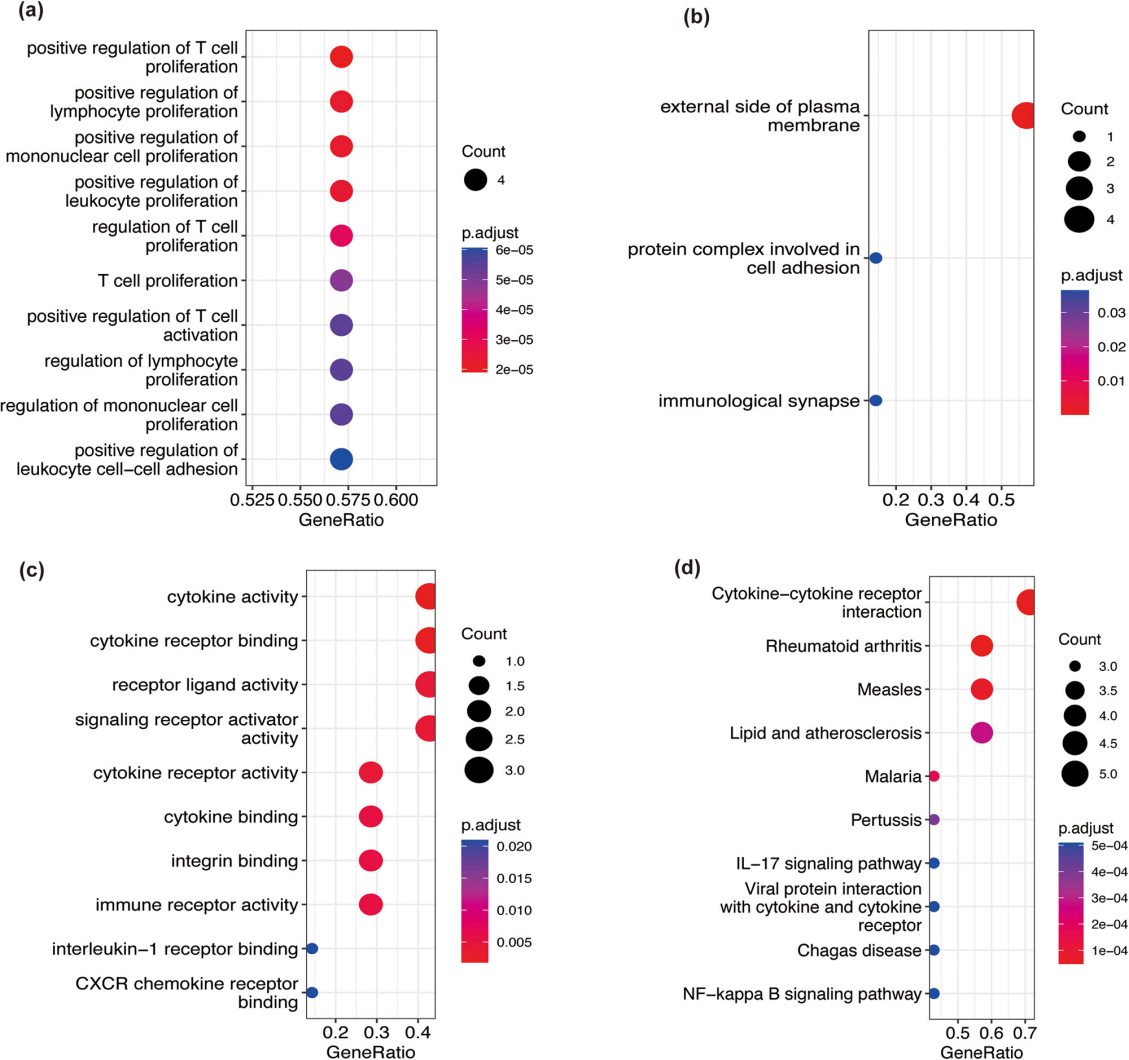
GO and KEGG analyses of IRHGs. The red and blue colors represent high and low adjusted p values, respectively. The size of the dot indicates the amount of enriched IRHGs. (a) The top 10 BP enrichment terms. (b) The top 10 CC enrichment terms. (c) The top 10 MF enrichment terms. (d) The enriched KEGG pathways.

IRHGs were subjected to GO and KEGG functional enrichment analyses. The IRHGs were enriched in different GO terms as follows: BP term, T-cell proliferation and lymphocyte proliferation (Figure 4a); CC term, external side of plasma membrane (Figure 4b); and MF term, cytokine activity (Figure 4c). KEGG pathway enrichment analysis revealed that IRHGs were mainly enriched in cytokine–cytokine receptor interaction, interleukin (IL)-17 signaling pathway, and nuclear factor-kappa B (NF-κB) signaling pathway (Figure 4d).

### 3.5 Profiling of immune cells associated with HF

**Figure 5:**
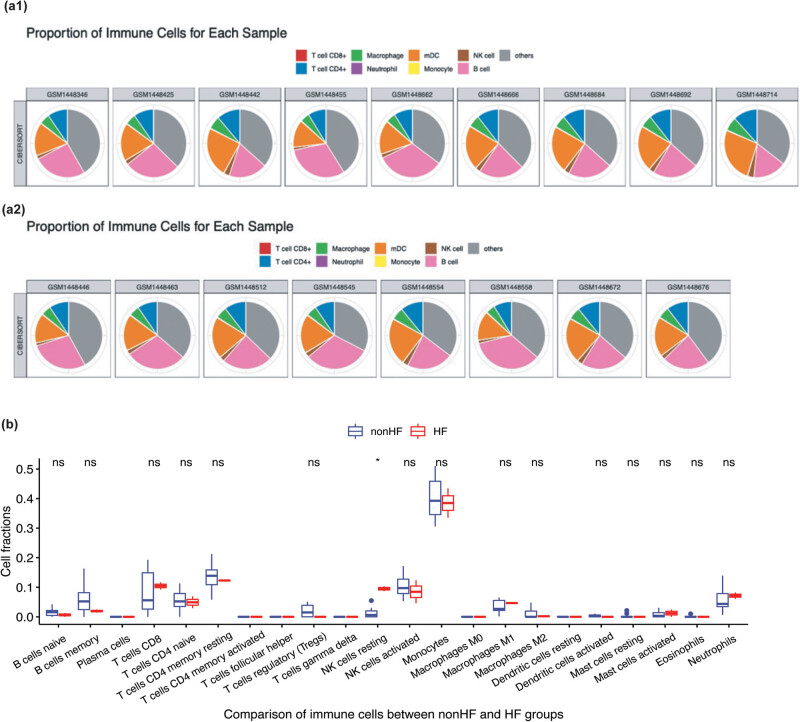
(a) The proportions of immune cells in the HF (a1) and non-HF groups (a2). (b) Comparative analysis of the fraction of immune cells between the HF and non-HF groups. Resting NK cells were significantly upregulated in the HF group. *p < 0.05.

Next, the abundance of the immune cells in the AMI samples was profiled using the CIBERSORT algorithm (Figure 5a1 and a2). Comparative analysis of the abundance of immune cells between the HF and non-HF groups revealed that the proportion of resting natural killer (NK) cells was significantly upregulated in the HF group (Figure 5b).

### 3.6 Validation of hub genes associated with HF

**Figure 6:**
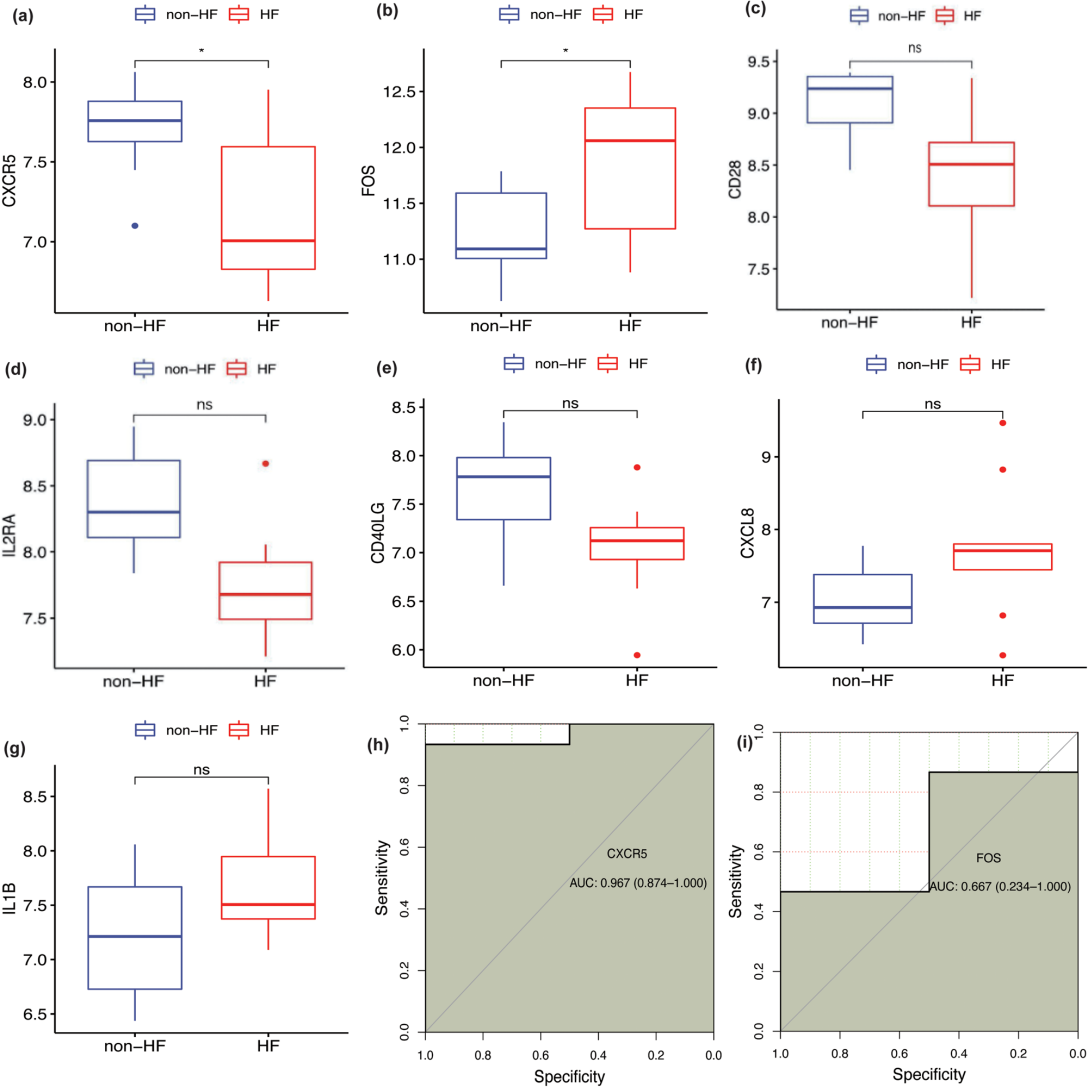
Profiling of seven IRHGs associated with HF and evaluation of the ability of *CXCR5* and *FOS* to predict long-term HF. (a–g) Comparative analysis of the expression levels of seven IRGs between the HF and non-HF groups. (h and i) ROC analysis of *CXCR5* and *FOS*. *p < 0.05.

To further assess the potential values of the seven IRHGs, the expression levels of IRHGs and their ability to predict long-term HF were determined. *CXCR5* and *FOS* were differentially expressed between the HF and non-HF groups (Figure 6a–g). Additionally, *CXCR5* (AUC = 0.967; 95% confidence interval = 0.874–1) and *FOS* (AUC = 0.667; 95% confidence interval = 0.234–1) could predict long-term HF (Figure 6h and i).

### 3.7 Mouse experiments

**Figure 7:**
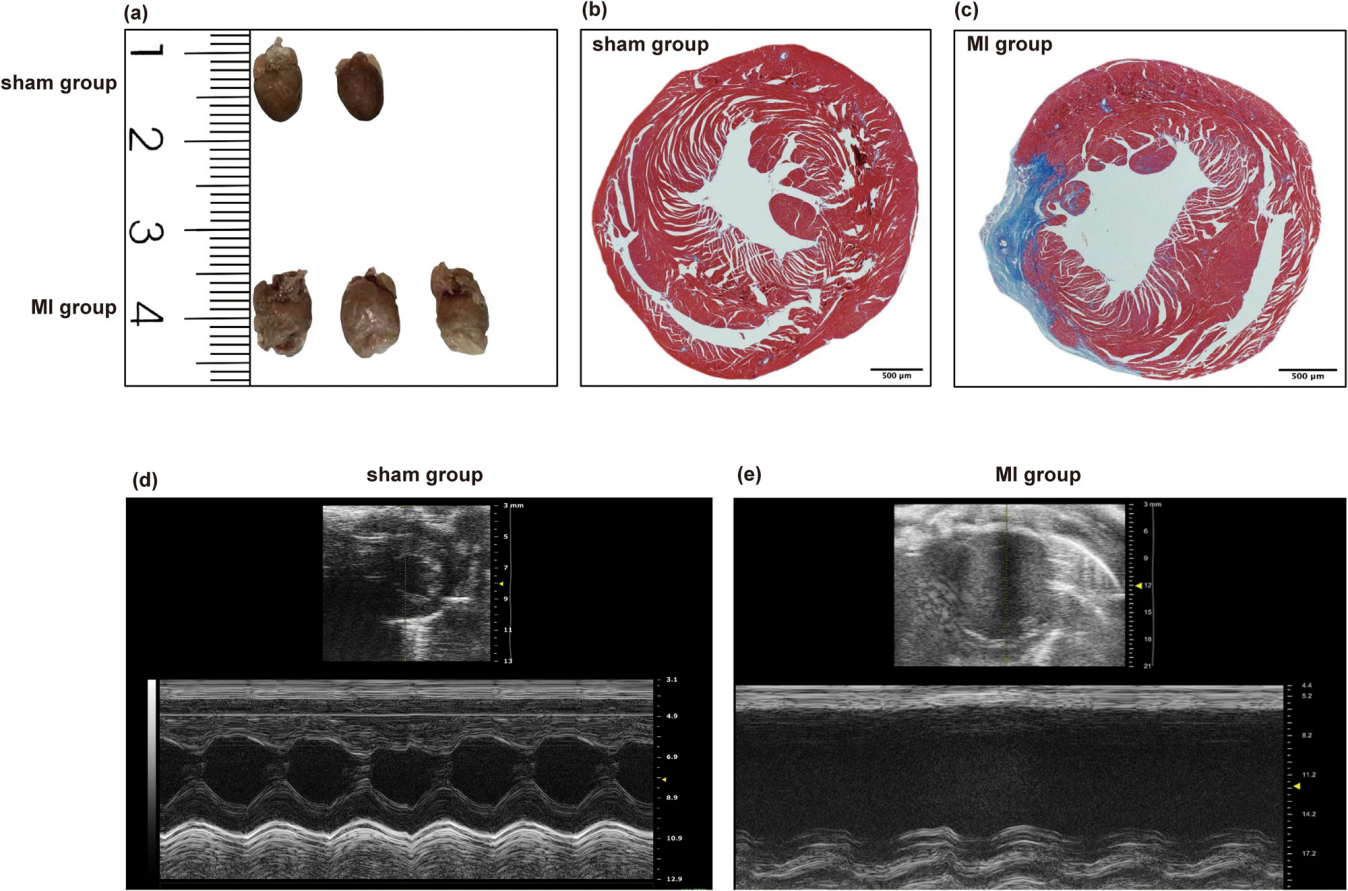
(a) The macrograph of hearts in the sham and MI group. (b and c) The representative images of cardiac sections from the sham and MI groups (scale bar = 500 μm) subjected to Masson’s staining. (d and e) M-mode echo-cardiograms of the sham and MI groups.

The heart size in the MI group was higher than that in the sham group (Figure 7a). The representative images of heart sections from the sham and MI groups subjected to Masson’s staining on day 60 post-surgery (scale bars = 500 μm) are presented in Figures 7b and c. Cardiac fibrosis was observed in the MI group (Figure 7c). The cardiac function of mice in the sham and MI groups was evaluated using M-mode echocardiography. The left ventricular movements in the MI group were significantly poor when compared with those in the sham group (Figures 7d and e).

**Figure 8:**
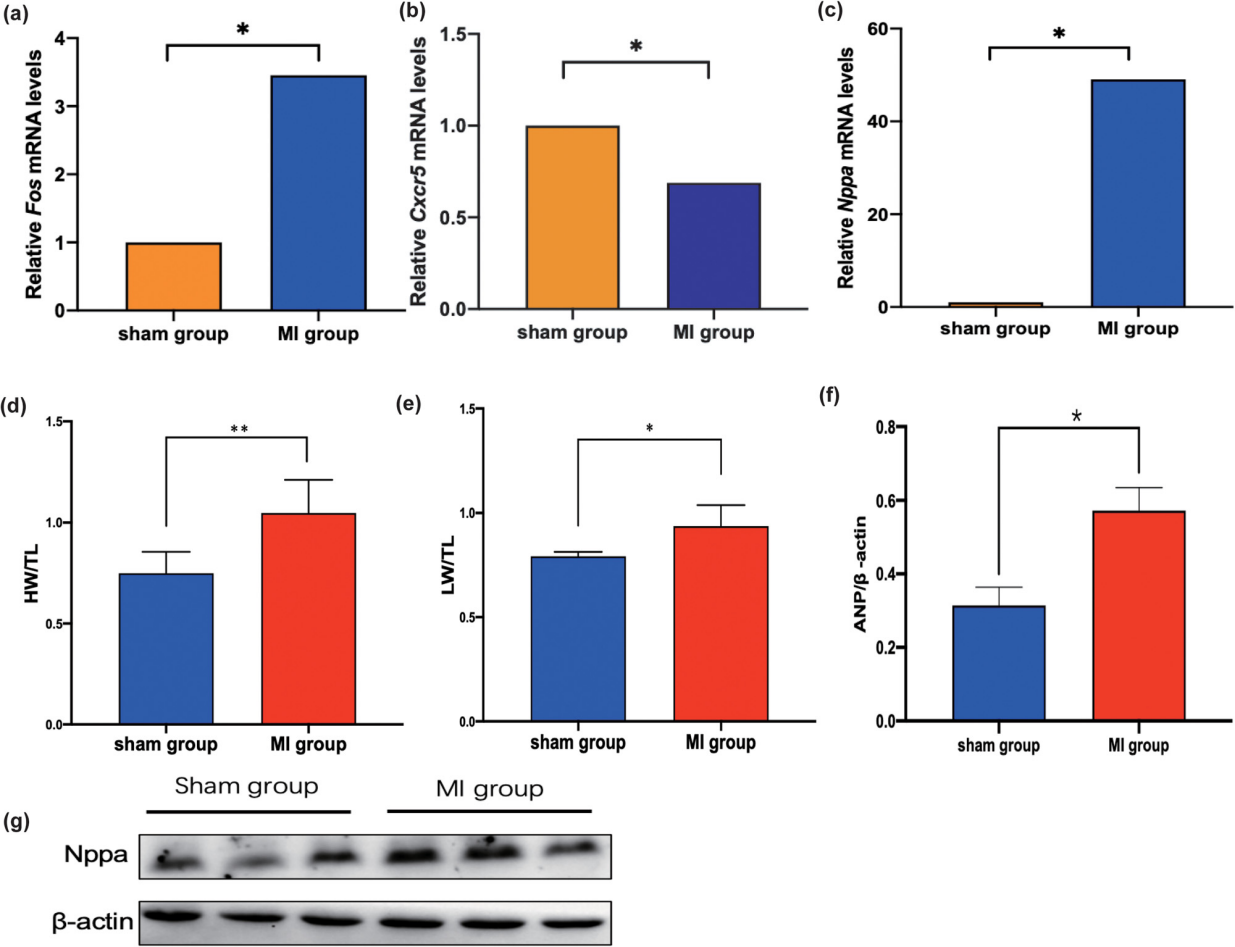
(a–c) The mRNA expression levels of *Fos*, *Cxcr5*, and *Nppa* in the sham (*n* = 5) and MI groups (*n* = 5). (d and e) The ratios of HW/TL and liver weight (LW)/TL in the sham (*n* = 5) and MI groups (*n* = 5). (f and g) The Nppa protein levels in the sham (*n* = 5) and MI groups (*n* = 5) were evaluated using western blotting. *p < 0.05; **p < 0.01.

The effects of HF post-AMI on the mRNA expression levels of *Cxcr5*, *Fos*, and atrial natriuretic factor (*Nppa*) were examined using qRT-PCR analysis. The *Fos* and *Nppa* mRNA levels in the MI group were upregulated when compared with those in the sham group. Meanwhile, the *Cxcr5* mRNA levels were downregulated in the MI group (Figure 8a–c) (*p* < 0.05). The heart weight (HW), lung weight (LW), and tibia length (TL) were measured in each mouse. The ratios of HW/TL and LW/TL were markedly different between the MI and sham groups (Figure 8d and e) (*p* < 0.05). Western blotting analysis revealed that the Nppa protein levels in the MI group were significantly higher than those in the sham group (Figure 8f and g) (*p* < 0.05). These results indicated that the genes identified in this study were associated with the development of HF post-AMI.

## 4 Discussion

This study revealed that *CXCR5* and *FOS* were differentially expressed between the HF and non-HF groups and could predict long-term HF. Additionally, functional enrichment analysis revealed that *CXCR5* and *FOS* were enriched in immune system processes associated with the progression of HF, including the IL17 and NF-κB signaling pathways. These novel biomarkers can complement current biomarkers for predicting long-term HF in patients with AMI and provide novel insights for understanding HF development. Previous studies have identified potential biomarkers for HF post-AMI, such as *FMN1*, *JDP2*, *LRRN3*, *FADS2*, *GPR15*, and *AK5* [9,10]. However, the correlation of these indicators with immune mechanisms has not been elucidated. This study revealed that the proportion of resting NK cells varied between the HF post-AMI and non-HF groups. Additionally, *CXCR5* and *FOS* were demonstrated to predict long-term HF. Furthermore, in vivo experiments were performed to validate the in silico findings. Thus, this study demonstrated the role of immune response in the development of HF and provided a scientific rationale for future studies on HF.

CXCR5, a chemokine receptor of CXCL13, is expressed on mature B cells and follicular T helper cells and regulates homeostatic lymphoid cell trafficking and homing to B-cell follicles within secondary lymphoid organs [22,23]. In combination with the members of the lymphotoxin/tumor necrosis factor family, CXCR5 and its ligand (CXCL13) promote the development and maintenance of secondary lymphoid organs. *Cxcr5*-deficient mice are reported to exhibit impaired B-cell follicle development [23,24]. Heinrichs et al. [25] reported that compared with those in wild-type mice, the post-MI infiltration levels of B cells were downregulated *Cxcr5*-deficient mice or in mice treated with anti-CXCL13 neutralizing antibody. Recent studies have demonstrated that B cells and antibodies can modulate inflammation and remodeling after MI [25]. Waehre et al. revealed that after 3 weeks of aortic banding, *Cxcr5* knockout (*Cxcr5* ^*−/−*^) mice exhibited left ventricular dilation when compared with wild-type mice. Additionally, the myocardial CXCR5 levels are upregulated in patients with HF [26].

One study examined CXCR5 expression in thrombus obtained from plaque rupture during MI and reported strong CXCR5 immunostaining of CXCR5 in the thrombus of patients with ST-elevation MI and unstable carotid lesion. Additionally, factors released from thrombin-activated platelets upregulated the levels of CXCR5 in monocytes [27]. At day 7 post-MI, MMP12 inhibitor upregulated CXCR5 [28]. Thus, the activation of immune responses is critical for the pathogenesis of MI and HF, such as promoting adverse cardiac remodeling and left ventricular disorder.

Palomer et al. reported that miR-146a overexpression downregulated the *FOS* mRNA and protein levels. MMP9 activity was suppressed upon miR-146a-mediated downregulation of the FOS-AP-1 pathway. HF is closely associated with the upregulation of MMP9 protein [29]. Xue et al. reported that the anti-cardiac fibrotic effect of miR-29b-3p is partly mediated by targeting FOS [30]. Thus, *FOS* is involved in the pathogenesis of cardiac fibrosis and HF.

Immune cells are reported to promote myocardial injury [31–33]. In particular, monocytes and macrophages in the infarcted heart can elicit inflammatory responses and promote left ventricular adverse remodeling and HF [32,34,35]. This study demonstrated that the proportions of resting NK cells were significantly upregulated in patients with HF. These findings improved our understanding of the novel mechanism underlying post-AMI HF.

KEGG pathway enrichment analysis revealed that IRHGs were enriched in the IL17 and NF-κB signaling pathways. The IL17 pathway, which is a potential therapeutic target for immune-mediated diseases, has been widely studied and demonstrated to be involved in the pathogenesis of cardiac diseases [36,37]. Compared with those in control subjects, the IL17 levels are upregulated in patients with HF [36,38]. IL17 promotes myocardium apoptosis and ischemic myocardium remodeling [39]. A previous study reported that IL17 promotes fibrosis, collagen generation, and apoptosis response and that these effects of IL17 were reversed upon treatment with neutralizing antibodies [37]. Il17 is upregulated during the pathogenesis of HF in mice through the NF-κB-dependent suppression of Atp2a2 and Cacna1c expression [36].

The transcription factor NF-κB is the downstream mediator of the IL17 signaling pathway [40]. The induction of NF-κB promotes the release of inflammatory cytokines, chemokines, and adhesion molecules from innate and adaptive immune cells. The subsequent inflammatory cascades adversely affect the cardiovascular system [41]. NF-κB, which mediates the pathological processes of various cardiovascular diseases, is reported to be involved in promoting inflammation, cell survival, cell differentiation, and cell proliferation [41], which contribute to the progression of cardiovascular diseases, such as HF, myocardial ischemia, hypertension, and atherosclerosis [41].

This study has several limitations. The sample size in this study was small, which may lead to biased conclusions owing to population differences. The CIBERSORT algorithm has several limitations. For example, the CIBERSORT algorithm can systematically underestimate or overestimate certain cell types, even though it is associated with a relatively lower estimation bias than other similar methods [16].

Our study successfully identified two IRHGs that were differentially expressed between the HF and non-HF groups. These genes could predict long-term HF. Thus, the findings of this study provided novel insights for understanding HF development.

## References

[1] Shioi T, Matsumori A, Kihara Y, Inoko M, Ono K, Iwanaga Y, et al. Increased expression of interleukin-1 beta and monocyte chemotactic and activating factor/monocyte chemoattractant protein-1 in the hypertrophied and failing heart with pressure overload. Circ Res. 1997;81:664–71. 10.1161/01.res.81.5.664 9351439

[2] Epelman S, Lavine KJ, Beaudin AE, Sojka DK, Carrero JA, Calderon B, et al. Embryonic and adult-derived resident cardiac macrophages are maintained through distinct mechanisms at steady state and during inflammation. Immunity. 2014;40:91–104. 10.1016/j.immuni.2013.11.019 PMC3923301 24439267

[3] Patel B, Bansal SS, Ismahil MA, Hamid T, Rokosh G, Mack M, et al. CCR2 + monocyte-derived infiltrating macrophages are required for adverse cardiac remodeling during pressure overload. JACC Basic Transl Sci. 2018;3:230–44. 10.1016/j.jacbts.2017.12.006 PMC6059350 30062209

[4] Laroumanie F, Douin-Echinard V, Pozzo J, Lairez O, Tortosa F, Vinel C, et al. CD4 + T cells promote the transition from hypertrophy to heart failure during chronic pressure overload. Circulation. 2014;129:2111–24. 10.1161/CIRCULATIONAHA.113.007101 24657994

[5] Nevers T, Salvador AM, Grodecki-Pena A, Knapp A, Velázquez F, Aronovitz M, et al. Left ventricular T-Cell recruitment contributes to the pathogenesis of heart failure. Circ Heart Fail. 2015;8:776–87. 10.1161/CIRCHEARTFAILURE.115.002225 PMC4512916 26022677

[6] Kallikourdis M, Martini E, Carullo P, Sardi C, Roselli G, Greco CM, et al. T cell costimulation. blockade blunts pressure overload-induced heart failure. Nat Commun. 2017;8:14680. 10.1038/ncomms14680 PMC5343521 28262700

[7] Martini E, Kunderfranco P, Peano C, Carullo P, Cremonesi M, Schorn T, et al. Single-Cell Sequencing of Mouse Heart Immune Infiltrate in Pressure Overload–Driven Heart Failure Reveals Extent of Immune Activation. Circulation. 2019;140:2089–107. 10.1161/CIRCULATIONAHA.119.041694 31661975

[8] Zhang Y, Bauersachs J, Langer H. Immune mechanisms in heart failure. Eur J Heart Fail. 2017;19(11):1379–89. 10.1002/ejhf.942 28891154

[9] Ma XD, Zhang QJ, Zhu HH, Huang KF, Pang WN, Zhang Q. Establishment and analysis of the lncRNA-miRNA-mRNA network based on competitive endogenous RNA identifies functional genes in heart failure. Math Biosci Eng. 2021;18(4):4011–26. 10.3934/mbe.2021201 34198423

[10] Shah Y, Verma A, Marderstein AR, White J, Bhinder B, Garcia Medina JS, et al. Pan-cancer analysis reveals molecular patterns associated with age. Cell Rep. 2021;37:110100. 10.1016/j.celrep.2021.110100 34879281

[11] Newman AM, Liu CL, Green MR, Gentles AJ, Feng W, Xu Y, et al. Robust enumeration of cell subsets from tissue expression profiles. Nat Methods. 2015;12:453–7. 10.1038/nmeth.3337 PMC4739640 25822800

[12] Huang C, Zhang C, Sheng J, Wang D, Zhao Y, Qian L, et al. Identification and validation of a tumor microenvironment-related gene signature in hepatocellular carcinoma prognosis. Front Genet. 2021;12:717319. 10.3389/fgene.2021.717319 PMC8662347 34899826

[13] Zheng XN, Zhou XH, Xu H, Jin D, Yang L, Shen BR, et al. A novel immune-gene pair signature revealing the tumor microenvironment features and immunotherapy prognosis of muscle-invasive bladder cancer. Front Genet. 2021;12:764184. 10.3389/fgene.2021.764184 PMC8664435 34899849

[14] Maciejak A, Kisiszek M, Michalak M, Tulacz D, Opolski G, Matlak K. Gene expression profiling reveals potential prognostic biomarkers associated with the progression of heart failure. Genome Med. 2015;7(1):26. 10.1186/s13073-015-0149-z PMC4432772 25984239

[15] Li XF, Li B, Jiang H. Identification of time-serious differentially expressed genes and pathways associated with heart failure post-myocardial infarction using integrated bioinformatics analysis. Mol Med Rep. 2019;19(6):5281–90. 10.3892/mmr.2019.10190 PMC6522961 31059043

[16] Gautier L, Cope L, Bolstad BM, Irizarry RA. Affy-analysis of Affymetrix GeneChip data at the probe level. Bioinformatics. 2004;20(3):307–15. 10.1093/bioinformatics/btg405 14960456

[17] Ritchie ME, Phipson B, Wu D, Hu YF, Law CW, Shi W, et al. Limma powers differential expression analyses for RNA-sequencing and microarray studies. Nucleic Acids Res. 2015;43(7):e47. 10.1093/nar/gkv007 PMC4402510 25605792

[18] Yu GC, Wang LG, Han YY, He QY. Cluster Profiler: An R package for comparing biological themes among gene clusters. OMICS. 2012;16:284–7. 10.1089/omi.2011.0118 PMC3339379 22455463

[19] Subramanian A, Tamayo P, Mootha VK, Mukherjee S, Ebert BL, Gillette MA, et al. Gene set enrichment analysis: A knowledge-based approach for interpreting genome-wide expression profiles. Proc Natl Acad Sci U S A. 2005;102(43):15545–50. 10.1073/pnas.0506580102 PMC1239896 16199517

[20] Cedric G. ggplot2: Elegant graphics for data analysis. J R Stat Soc Ser A Stat Soc. 2011;174:678–9.

[21] Zhang Y, Li C, Meng H, Guo DQ, Zhang Q, Lu WJ, et al. BYD ameliorates oxidative stress-induced myocardial apoptosis in heart failure post-acute myocardial infarction via the P38 MAPK-CRYAB signaling pathway. Front Physiol. 2018;9:505. 10.3389/fphys.2018.00505 PMC5951999 29867551

[22] Bunse M, Pfeilschifter J, Bluhm J, Zschummel M, Joedicke JJ, Wirges A, et al. CXCR5 CAR-T cells simultaneously target B cell non-Hodgkin’s lymphoma and tumor-supportive follicular T helper cells. Nat Commun. 2021;12:240. 10.1038/s41467-020-20488-3 PMC7801647 33431832

[23] Förster R, Mattis AE, Mremmer E, Wolf E, Brem G, Lipp M. A putative chemokine receptor, BLR1, directs B cell migration to defined lymphoid organs and specific anatomic compartments of the spleen. Cell. 1996;87:1037–47. 10.1016/s0092-8674(00)81798-5 8978608

[24] Muller G, Hopken UE, Lipp M. The impact of CCR7 and CXCR5 on lymphoid organ development and systemic immunity. Immunol Rev. 2003;195:117–35. 10.1034/j.1600-065x.2003.00073.x 12969315

[25] Heinrichs M, Ashour D, Siegel J, Büchner L, Wedekind G, Heinze KG, et al. The healing myocardium mobilizes a distinct B-cell subset through a CXCL13-CXCR5-dependent mechanism. Cardiovasc Res. 2021;117(13):2664–76. 10.1093/cvr/cvab181 34048536

[26] Waehre A, Halvoresn B, Yndestad A, Husberg C, Sjaastad I, Nygård S, et al. Lack of chemokine signaling through CXCR5 causes increased mortality, ventricular dilation and deranged matrix during cardiac pressure overload. PLoS One. 2011;6(4):e18668. 10.1371/journal.pone.0018668 PMC3078912 21533157

[27] Halvorsen B, Smedbakken LM, Michelsen AE, Skjelland M, Bjerkeli V, Sagen EL, et al. Activated platelets promote increased monocyte expression of CXCR5 through prostaglandin E2-related mechanisms and enhance the anti-inflammatory effects of CXCL13. Atherosclerosis. 2014;234(2):352–9. 10.1016/j.atherosclerosis.2014.03.021 24732574

[28] Lyer RP, Patterson NL, Zouein FA, Ma YG, Dive V, Lindesy ML, et al. Early matrix metalloproteinase-12 inhibition worsens post-myocardial infarction cardiac dysfunction by delaying inflammation resolution. Int J Cardiol. 2015;185:198–208. 10.1016/j.ijcard.2015.03.054 PMC4406852 25797678

[29] Palomer X, Capdevila-Busquets E, Botteri G, Davidson MM, Rodríguez C, Martínez-González J, et al. miR-146a targets Fos expression in human cardiac cells. Dis Model Mech. 2015;8(9):1081–91. 10.1242/dmm.020768 PMC4582106 26112171

[30] Xue YL, Fan XF, Yang RB, Jiao YY, Li Y. miR-29b-3p inhibits post-infarct cardiac fibrosis by targeting FOS. Biosci Rep. 2020;40(9):BSR20201227. 10.1042/BSR20201227 PMC7468097 32812641

[31] Heidt T, Courties G, Dutta P, Sager HB, Sebas M, Iwamoto Y, et al. Differential contribution of monocytes to heart macrophages in steady-state and after myocardial infarction. Circ Res. 2014;115:284–95. 10.1161/CIRCRESAHA.115.303567 PMC4082439 24786973

[32] Nahrendorf M. Myeloid cell contributions to cardiovascular health and disease. Nat Med. 2018;24:711–20. 10.1038/s41591-018-0064-0 PMC7301893 29867229

[33] Swirski FK, Nahrendorf M. Cardioimmunnology: The immune system in cardiac homeostasis and disease. Nat Rev Immunol. 2018;18:733–44. 10.1038/s41577-018-0065-8 30228378

[34] Aurota AB, Porrello ER, Tan W, Mahmoud AI, Hill JA, Bassel-Duby R, et al. Macrophages are required for neonatal heart regeneration. J Clin Invest. 2014;124:1382–92. 10.1172/JCI72181 PMC3938260 24569380

[35] Bajpai G, Schneider C, Wong N, Bredemeyer A, Hulsmans M, Nahrendorf M, et al. The human heart contains distinct macrophage subsets with divergent origins and functions. Nat Med. 2018;24:1234–45. 10.1038/s41591-018-0059-x PMC6082687 29892064

[36] Xue GL, Li DS, Wang ZY, Liu Y, Yang JM, Li CZ, et al. Interleukin-17 upregulation participates in the pathogenesis of heart failure in mice via NF-κB-dependent suppression of SERCA2a and Cav1.2 expression. Acta Pharmacol Sin. 2021;42(11):1780–9. 10.1038/s41401-020-00580-6 PMC8563866 33589793

[37] Simon1 T, Taleb S, Danchin N, Laurans L, Rousseau B, Cattan S, et al. Circulating levels of interleukin-17 and cardiovascular outcomes in patients with acute myocardial infarction. Eur Heart J. 2013;34(8):570–7. 10.1093/eurheartj/ehs263 22956509

[38] Rahmati Z, Amirzargar AA, Saadati S, Rahmani F, Mahmoudi MJ, Rahnemoon Z, et al. Association of levels of interleukin 17 and T-helper 17 count with symptom severity and etiology of chronic heart failure: A case-control study. Croat Med J. 2018;59:139–48. 10.3325/cmj.2018.59.139 PMC6139427 30203627

[39] Zhou SF, Yuan J, Liao MY, Xia N, Tang TT, Li JJ, et al. IL-17A promotes ventricular remodeling after myocardial infarction. J Mol Med (Berl). 2014;92:1105–16. 10.1007/s00109-014-1176-8 24965614

[40] Iwakura Y, Ishigame H, Saijo S, Nakae S. Functional specialization of interleukin-17 family members. Immunity. 2011;34:149–62. 10.1016/j.immuni.2011.02.012 21349428

[41] Cheng WJ, Cui C, Liu G, Ye CJ, Shao F, Bagchi AK, et al. NF-κB, A Potential therapeutic. Target in Cardiovascular Diseases. Cardiovasc Drugs Ther. 2023;37(3):571–84. 10.1007/s10557-022-07362-8 35796905

